# The Treatment of Varicocele

**Published:** 1891-09

**Authors:** 


### THE TREATMENT OF VARICOCELE.

According to Dr. Landerer, the extirpation or obliteration of varicose scrotal veins is apt to be followed by relapses. In one of his cases of recurrence he obtained an excellent result by letting his patient wear a hernia truss with movable pad, according to the method of Ravoth. One and a half years after application of the truss, the varix had disappeared completely, and after wearing it for a year longer it was left off, and since eight years there had been no return of the varicocele. An equally successful result was obtained in three other cases. The truss probably acts as an artificial substitute for the valves of the veins which have either completely disappeared in the varicosities or are in a rudimentary condition. This procedure is also applicable to varices of the lower extremities. For this purpose the author makes use of an apparatus consisting of a curved spring and a pad filled with water, which is made to press directly upon the saphena vein. He has employed this truss in eighty cases with very satisfactory results. Its application is much more agreeable to the patient than bandaging, and it is much cheaper than an elastic stocking. Under the use of the apparatus a reduction in the size of the limb has been observed, but the treatment must be regarded as palliative rather than curative.—*Deut. Medizinal-Zeitung.—Inter. Jour. Surg.*

